# Quality improvement of surface triangular mesh using a modified Laplacian smoothing approach avoiding intersection

**DOI:** 10.1371/journal.pone.0184206

**Published:** 2017-09-08

**Authors:** Tiantian Liu, Minxin Chen, Yu Song, Hongliang Li, Benzhuo Lu

**Affiliations:** 1 Department of Mathematics, Soochow University, Suzhou 215006, China; 2 State Key Laboratory of Scientific and Engineering Computing, National Center for Mathematics and Interdisciplinary Sciences, Academy of Mathematics and Systems Science, Chinese Academy of Sciences, Beijing 100190, China; 3 Microsystem & Terahertz Research Center, China Academy of Engineering Physics, Chengdu, China; Nankai University, CHINA

## Abstract

We present a systematic procedure to improve the qualities of triangular molecular surface meshes and at the same time preserve the manifoldness. The procedure utilizes an algorithm to remove redundant points having three or four valences and another algorithm to smooth the mesh using a modified version of Laplacian method without causing intersecting triangles. This approach can be effectively applied to any manifold surface meshes with arbitrary complex geometry. In this paper, the tested meshes are biomolecular surface meshes exhibiting typically highly irregular geometry. The results show that the qualities of the surface meshes are greatly improved and the manifoldness of the surface meshes are preserved. Compared with the original meshes, these improved molecular surface meshes can be directly applied to boundary element simulations and generation of body-fitted volume meshes using Tetgen. The procedure has been incorporated into our triangular molecular surface mesh generator, TMSmesh 2.0. It can be also used as a standalone program and works together with any other surface triangular mesh generator to obtain qualified manifold mesh. The package is downloadable at https://doi.org/10.6084/m9.figshare.5346169.v1 and can be run online at http://www.xyzgate.com.

## Introduction

Surface mesh generation arises in many applications, such as numerical simulation, computer visualization and geometry processing. Most computational applications involve triangulation of a complex surface geometry, especially in computational biology. Molecular surface plays an important role in computational biology, such as protein folding, structure prediction, docking and implicit solvent modeling. Recent developments in realistic mathematical modeling and numerical simulation of biomolecular systems raise new demands for qualified, stable, and efficient surface meshing, especially in implicit-solvent modeling [1]. Many triangulated meshes are generated by scanning devices or by isosurfacing implicit representations. However, it is not easy to generate high-quality mesh for complex surface geometry in such processes, especially if automated. Low-quality triangular meshes can undermine the order of accuracy or even cause non-convergence in numerical computations. In this case, it is desirable to build high-quality meshes from those low-quality meshes before performing any numerical simulation. [2]

Mesh quality refers to the faithfulness, manifoldness and uniformness. Faithfulness is measured by how accurately the surface mesh preserves the original geometry and topology, such as surface area, volume and curvature of the referenced surface. Faithfulness related to the accuracy of the numerical simulation and geometry processing. Manifoldness of a surface mesh means that each point on the surface has a neighborhood which is homeomorphic to a disk in a real plane. Non-manifold surface mesh brings difficulties in generating corresponding surface conforming volume mesh that is an essential prerequisite for successful finite element method computations. One of the typical non-manifold errors in surface meshes is intersecting triangles. It is an important issue that preserving the manifoldness of the original surface meshes in the process of improvements of mesh qualities. Uniformness includes the triangle shape, regularity, complexity and so on. The quality of triangles in surface mesh is crucial for robustness of boundary element method (BEM) and finite element method (FEM) computations. In some numerical analysis, such as finite element analysis, regular or qualified mesh is always required. The mesh size is related to the complexity of the mesh. Generally, more sampling points capture more surface details. But more sampling points increase the complexity of the mesh, which increases the difficulty of numerical simulation. Therefore, meshes can be improved with respect to any number of quality metrics including shape, size, manifoldness, solution error or combinations of these. The improvement process is called remeshing. [3] There are two fundamental remeshing approaches, parameterization techniques [4–10] and mesh adaptation strategies [11–15]. In the context of the parameterization-based remeshing, the initial surface is parameterized onto a surface in 2D, the 2D surface is meshed by the standard mesh generation method and the new mesh is projected back to the original surface. The existing parameterization method is classified to linear method [16–21], non-linear method [22–24] and hybrid method [25, 26]. The parameterization-based methods can work even for coarse resolution remeshing, but the computation is expensive and the result is sensitive to the specific parameterization. The mesh adaptation method treats the surface as a set of points in space and operate directly on the surface. The strategies use local mesh modifications to improve the quality of the input surface mesh or adapt the mesh to a given mesh size criterion. Typical techniques for local mesh modifications combine vertex smoothing, vertex insertion/deletion, vertex shift, edge split, edge flip, edge collapse and so on. [10, 27–30]

Recently, we have developed a method, TMSmesh [31, 32] and its improved version, TMSmesh 2.0 [33], to generate molecular surface mesh for large biomolecules. TMSmesh 2.0 succeeds to generate manifold surface meshes for biomolecules comprised of more than one million atoms efficiently. The generated surface mesh preserves the original molecular surface features and properties (topology, surface area, enclosed volume, local curvature and so on) [34]. However, the mesh quality in the aspects of uniformness and smoothness are not guaranteed. There are some redundant points and some singular triangles in the meshes generated by TMSmesh 2.0. In boundary element and finite element simulation for some large biomolecules, the uniformness of the surface mesh is required to be improved to make the numerical simulation more robust and accurate. The faithfulness of the surface mesh to the original geometric features are critical in getting reliable simulation results from BEM and FEM computation in continuum molecular simulations. The parameterization-based methods contain the process of resampling, so these automated operation can not guarantee the fidelity of improved meshes to the original surface. And the complex geometry and topology of the large biomolecules will increase the difficulty of parameterization dramatically. There are some packages using mesh adaption strategies to improve mesh qualities, but few of them can preserve manifold meshes for complex surfaces. Therefore, in this work, we develop a package named SMOPT to improve the mesh quality directly on the surface and perform a series of local modifications on the mesh. The qualities of the new resulted meshes are improved greatly over the original surface meshes and avoid causing non-manifold errors, such as intersecting triangles. The program SMOPT has been incorporated into TMSmesh 2.0. It can be also used as a standalone program and works together with any other surface triangular mesh generator to obtain qualified manifold mesh.

In the next Section, the method used to improve the surface mesh quality is introduced. Some examples, analysis and applications are presented in the results section. The final section, Conclusion, gives some concluding remarks.

## Materials and methods

In this section, we describe the mesh generator TMSmesh 2.0 and the improvement procedure SMOPT to improve the quality of the triangular mesh generated by TMSmesh 2.0.

### 0.1 TMSmesh 2.0

TMSmesh 2.0 is an algorithm for manifold triangular meshing of Gaussian molecular surfaces. [31–33] The Gaussian surface is defined as a level set of the summation of Gaussian kernel functions:

$$\begin{array}{c} \{\vec{x}\in R^{3},\phi (\vec{x})=c\}, \end{array}$$ (1)

where

$$\begin{array}{c} \phi (\vec{x})=\sum_{i=1}^{N}e^{-d(∥\vec{x}-{\vec{x}}_{i}{∥}^{2}-r_{i}^{2})}, \end{array}$$ (2)

${\vec{x}}_{i}$ and *r*_*i*_ are the location and radius of the *i*th atom, *N* is the number of atoms in the molecule. *d* is the decay rate of the Gaussian kernel. When *d* decreases, the molecules will show more geometrical details. *c* is the isovalue and it controls the volume enclosed by the Gaussian surface [34].

The algorithm of TMSmesh 2.0 contains two stages, the first stage is an adaptive estimation and division process. The Gaussian surface is approximated by piecewise trilinear surface within controllable error. The second stage is to partition each piece of trilinear surface into single-valued patches along x, y, z directions by tracing along the fold curves. Then each single-valued patch is triangulated by the ear clipping algorithm. TMSmesh 2.0 succeeds to generate surface meshes for biomolecules comprised of more than one million atoms, and all the generated meshes are manifold mesh preserving the original detailed geometry of molecular surface. The sampled points in TMSmesh 2.0 are distributed according to the curvature and geometric features of molecular surface adaptively. In the regions with dramatic changing shapes, the density of the sampled points is high. It may result in some very close points in the mesh, which will bring difficulties to generate body-fitted volume mesh. And the very close points may cause singularity in numerical simulations for continuum modeling of biomolecules. Therefore, some methods are required to delete the redundant points and improve the mesh quality without changing the geometric features of molecules.

### 0.2 Algorithm to delete redundant points

The redundant points are chosen from the vertices whose valence is three or four. Here, valence is the number of the adjacent vertices connected by edges.

**Fig 1:**
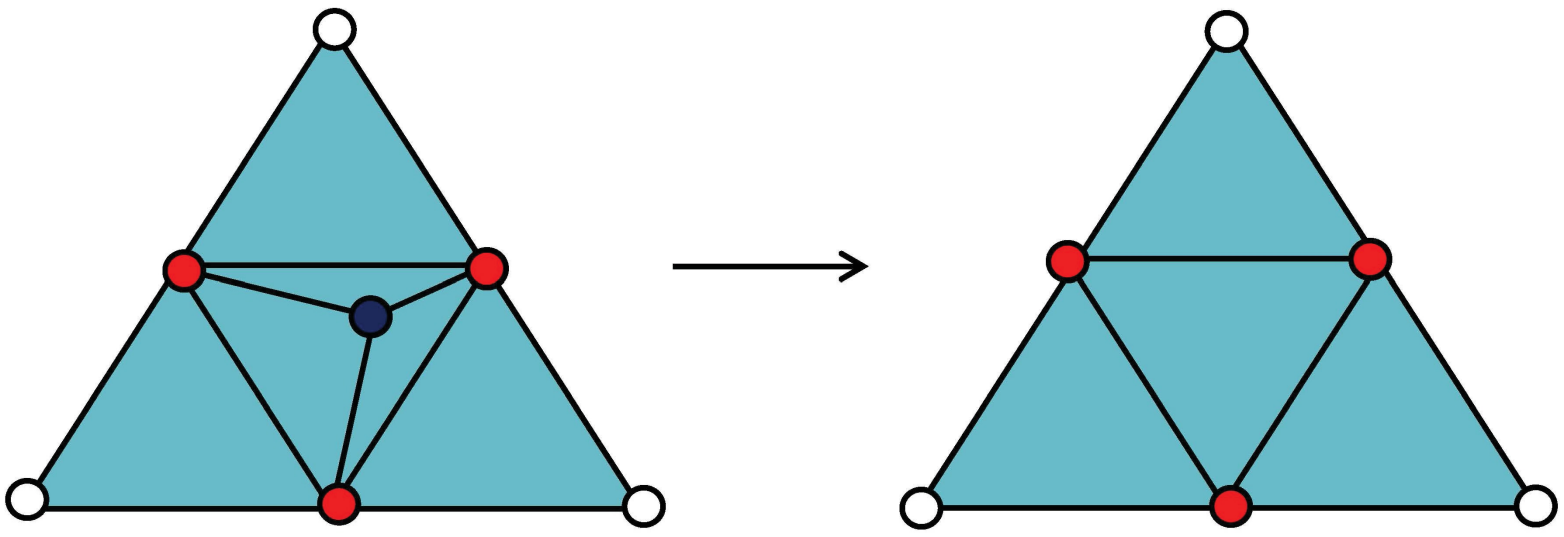
The black point is considered as the redundant point when it has a valence of three and is almost on the plane formed by its 1-ring neighborhoods in red color.

**Fig 2:**
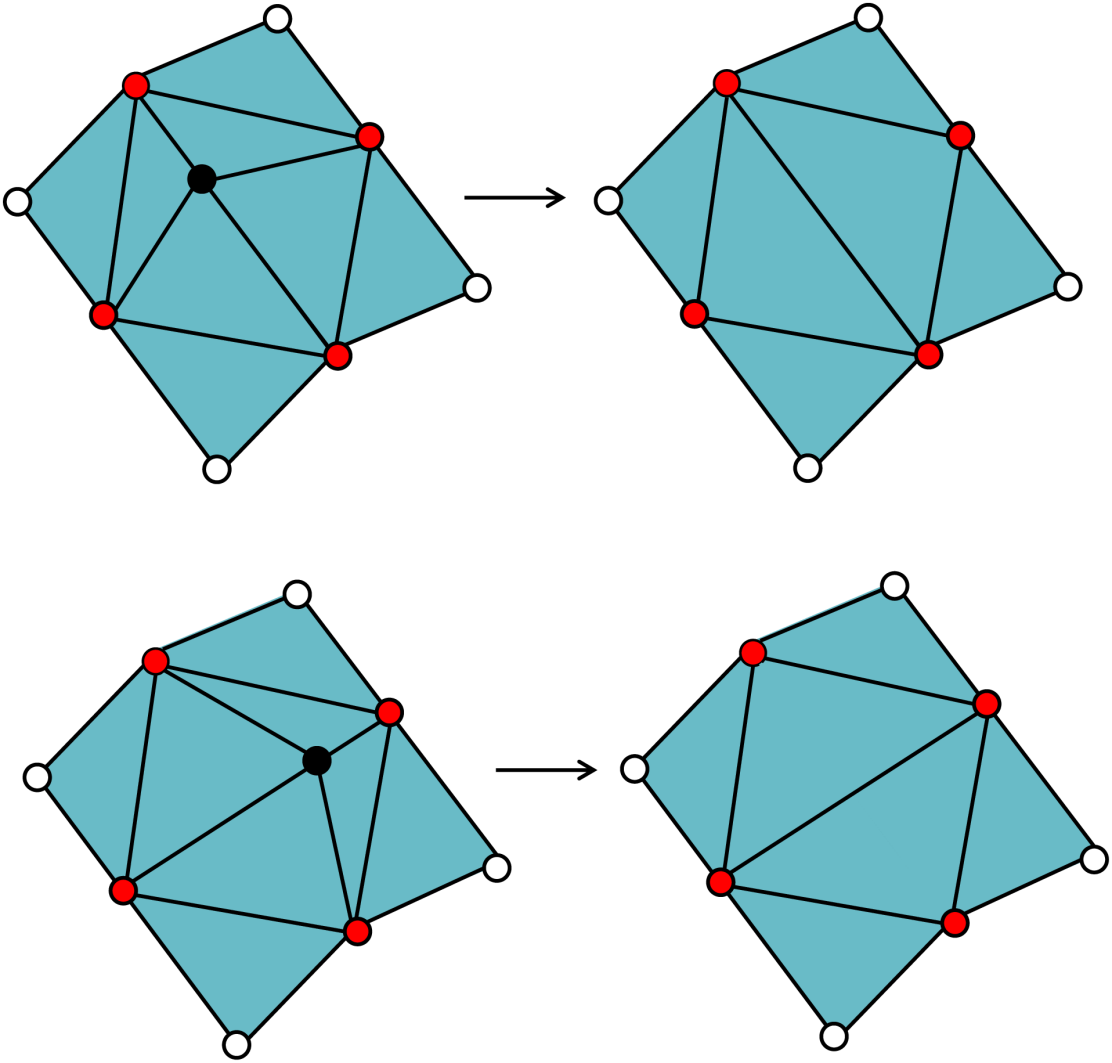
The redundant point whose valence is four. The black point is redundant point. The red points are 1-ring neighborhoods of the black point. The figure shows two cases that the black point is near two different diagonals.

Fig 1 shows the redundant condition that the valence of the point is three. When the black point is or almost on the plane formed by its three red connected neighboured points, the black point is considered as a redundant one. In this situation, the black point should be deleted. Meanwhile, the elements and the edges whose vertices contain the black point should also be deleted. Fig 2 shows the case of redundant vertex with a valence of four. When the black point is almost on one of two diagonals of the polygon formed by the four red connected neighboured points, the black point is considered as a redundant one. In this case, the black vertex, the elements and the edges whose vertices contain the black vertex should be deleted, and the diagonal on which the black vertex located should be added as a new edge. Then we can get two new triangles to replace the original four triangles containing the redundant vertex. Algorithm 1 shows the whole process to delete the redundant vertices.

The valence three and four cases are easy to handle. When the valence is three, we can delete the point directly. When the valance is four, we delete the point and re-triangulate the resulted polygon with four vertices. For this two simple cases, the deletion and re-triangulate processes will not lead to intersections, because these operations do not add any new planes. But for the cases with higher number of valence, after deleting the redundant vertex, we should re-triangulate the resulted polygon composed of the neighborhood triangles of the deleted vertex. It is hard to guarantee no intersection, especially when this polygon is not flat. Therefore, in these cases, we prefer to change the position of the vertices with more than four valence rather than delete.

**Algorithm 1** Delete the redundant points.

**Input:** vertices in the mesh and the connection relation of each vertex.

 *i* = 1

 *err* = 1*e* − 4

 **while** *i* ≤ num of vertices **do**

  **if** valence of vertex *i* is 3 **then**

   *n*_1_, *n*_2_, *n*_3_ are the points on the neighbour loop of vertex *i*

   *P* is the plane formed by *n*_1_, *n*_2_, *n*_3_

   **if** distance(*i*, *P*) < *err* **then**

    delete vertex *i*

    delete the triangles whose vertices contain *i*

    insert the new triangle formed by *n*_1_, *n*_2_, *n*_3_

   **end if**

  **end if** valence of vertex *i* is 4 **then**

   *n*_1_, *n*_2_, *n*_3_, *n*_4_ are the points on the neighbour loop of vertex *i*

   *n*_1_*n*_3_ and *n*_2_*n*_4_ are the diagonals of the neighbour loop

   **if** distance(*i*, *n*_1_*n*_3_) < *err* **then**

    delete vertex *i*

    delete the triangles whose vertices contain *i*

    insert two new triangles formed by *n*_1_, *n*_3_, *n*_4_ and *n*_1_, *n*_2_, *n*_3_

   **else if** distance(*i*, *n*_2_*n*_4_) < *err* **then**

    delete vertex *i*

    delete the triangles whose vertices contain *i*

    insert two new triangles formed by *n*_1_, *n*_2_, *n*_4_ and *n*_2_, *n*_3_, *n*_4_

   **end if**

  **end if**

 **end while**

### 0.3 Mesh smoothing

An inherent problem of the automated triangulated surface mesh generation is that the resulted polygonal surface appears faceted and may contains singular triangles with very small angles. The original Gaussian surface of the biomolecule is smooth. But the generated mesh can make the approximated surface rough. And the singular triangles may cause difficulties in BEM computation and corresponding body-fitted volume mesh generation. Therefore, smoothing method is necessary for improving the qualities of surface meshes.

Laplacian smoothing is an algorithm to smooth a triangular surface mesh. [35, 36] The Laplacian smoothing technique changes the position of nodes without modifying the topology of the mesh. In the process of Laplacian smoothing, each vertex is move to a new position defined by taking the average position of its neighbors:

$$\begin{array}{c} p_{i}=\frac{1}{N_{i}}\sum_{j=1}^{N_{i}}q_{j} \end{array}$$ (3)

where *N*_*i*_ is the number of the adjacent vertices of the *i*th node, *q*_*j*_ is the position of the *j*th adjacent vertex. To control the rate of smoothing, the current position *q*_*i*_ is also included in the calculation of the new position *p*_*i*_:

$$\begin{array}{c} p_{i}=\left(1-\beta \right)q_{i}+\frac{\beta }{N_{i}}\sum_{j=1}^{N_{i}}q_{j} \end{array}$$ (4)

where *β* is the parameter to control the rate of smoothing. Usually, *β* takes a value between [0, 1]. However, if the geometrical characters are very complex in a region, the Laplacian smoothing may cause self-intersection. To ensure manifoldness, we proposed the modified version of Laplacian smoothing as follows. For each vertex, initially, *β* is chosen to be 1 and the vertex is moved to a new position through Eq (4). Then we check whether this movement causes intersection near this vertex. And if it does result in intersecting triangles, redo this movement by Eq (4) using half *β*. This process is repeated until the new position of current vertex does not bring intersecting triangles. And in each time of checking intersecting triangles, we only need to check whether there is an edge intersecting a triangle in the vicinity of the current moved vertex.

**Fig 3:**
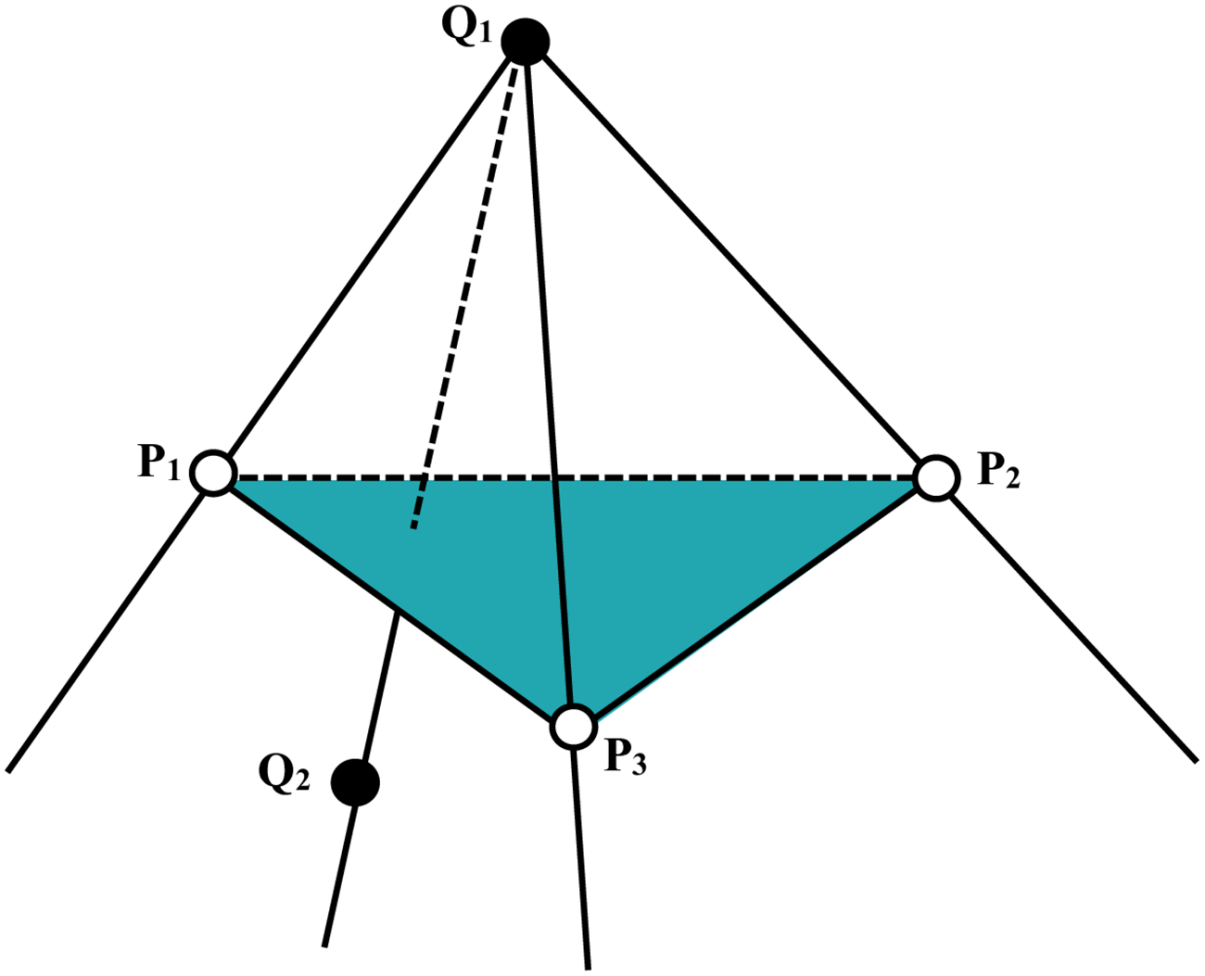
Diagram of an edge intersects with a triangle.

The key to detect self-intersection is to decide whether an edge is intersecting with a triangle. Fig 3 shows the case that an edge intersects with a triangle. In Fig 3, *P*_1_*P*_2_*P*_3_ is a triangle and *Q*_1_*Q*_2_ is an edge. If *Q*_2_ is in the shadow of *P*_1_*P*_2_*P*_3_ blocking light from the source *Q*_1_, the edge *Q*_1_*Q*_2_ intersect with the triangle *P*_1_*P*_2_*P*_3_. In other words, if *Q*_1_*Q*_2_ intersect with *P*_1_*P*_2_*P*_3_, following four conditions should be satisfied:

- the point *Q*_2_ and *P*_3_ are on the same side of the plane *Q*_1_*P*_1_*P*_2_
- the point *Q*_2_ and *P*_1_ are on the same side of the plane *Q*_1_*P*_2_*P*_3_
- the point *Q*_2_ and *P*_2_ are on the same side of the plane *Q*_1_*P*_3_*P*_1_
- the point *Q*_1_ and *Q*_2_ are on different sides of the plane *P*_1_*P*_2_*P*_3_.

Algorithm 2 shows the whole process to detect whether an edge intersects with a triangle.

**Algorithm 2** The algorithm to decide whether an edge intersects with a triangle.

**Input:** three vertices of a triangle *P*_1_, *P*_2_, *P*_3_ and two endpoints of an edge *Q*_1_, *Q*_2_

 $\vec{n_{1}}=\vec{Q_{1}P_{1}}\times \vec{Q_{1}P_{2}}$

 **if** dot_product($\vec{Q_{1}P_{3}},\vec{n_{1}}$)*dot_product($\vec{Q_{1}Q_{2}},\vec{n_{1}}$)< 0 **then**

  return False (False means no intersection and True means that the edge intersects with the triangle)

 **else**

  $\vec{n_{1}}=\vec{Q_{1}P_{2}}\times \vec{Q_{1}P_{3}}$

  **if** dot_product($\vec{Q_{1}P_{1}},\vec{n_{1}}$)*dot_product($\vec{Q_{1}Q_{2}},\vec{n_{1}}$)< 0 **then**

   return False

  **else**

   $\vec{n_{1}}=\vec{Q_{1}P_{3}}\times \vec{Q_{1}P_{1}}$

   **if** dot_product($\vec{Q_{1}P_{2}},\vec{n_{1}}$)*dot_product($\vec{Q_{1}Q_{2}},\vec{n_{1}}$)< 0 **then**

    return False

   **else**

    $\vec{n_{1}}=\vec{P_{1}P_{1}}\times \vec{P_{1}P_{3}}$

    **if** dot_product $\vec{Q_{1}P_{1}},\vec{n_{1}}$)*dot_product($\vec{Q_{2}P_{1}},\vec{n_{1}}$)> 0 **then**

     return False

    **else**

     return True

    **end if**

   **end if**

  **end if**

 **end if**

For large molecules, the amount of points and triangles are huge. If we do smoothing for all points every round, the computation is expensive. To reduce the computation complexity, we use a screening method. Every round doing the modified Laplacian smoothing, we just choose the vertices and the neighbored vertices of the low-quality triangles (the triangles with low edge ratio or containing tiny angles). The detailed algorithm is shown in algorithm 3. Firstly, we do modified Laplacian smoothing for all the points once. Then we find out all the triangles with low-quality. The vertices and the neighbored vertices of these low-quality triangles are considered as the target points which need to be smoothed next round. And we do smoothing to these points several rounds, usually three or four rounds. After this, we will find out the new irregular triangles from the targets we got last round. From the irregular triangles, we can get the new target points which should be smoothed next round. Repeating in this manner, the points which need to be smoothed are getting fewer and fewer. In this way, we don’t need to do modified Laplacian smoothing for all the points in the mesh every round, which reduces the computational cost greatly. When the quality of the mesh satisfies preset goal, the smoothing process is ended. Usually, we control the number of rounds of doing modified Laplacian smoothing for irregular triangles. In our program, the default rounds is set to 100. However, in most cases, the smoothing is convergent before reaching 100 rounds.

**Algorithm 3** The algorithm of mesh smoothing using modified Laplacian method.

**for** each point *P* in the initial mesh **do**

 do modified Laplacian smoothing to *P*

**end for**

let empty set *L* be a temporary point set

**for** each triangle *T* in the latest mesh **do**

 **if** the quality of *T* is required to be improved **then**

  add the vertices of *T* and all the neighbour vertices of *T* to the point set *L*

 **end if**

**end for**

**while** TRUE **do**

 do modified Laplacian smoothing to the point in point set *L* several rounds repeatedly construct a new temporary point set *L*′

 find all the singular triangles in *L* and add the vertices of the triangles and the neighbored vertices to *L*′

 set *L* = *L*′

 **if** rounds of doing modified Laplacian smoothing exceeds presupposed number

 **then**

  exit

 **end if**

**end while**

## Results

For large molecules, some surface meshes generated by TMSmesh 2.0 can not be directly used to boundary element method (BEM) simulation or generation of body-fitted volume mesh due to singular triangles with tiny angles or very short edges. The algorithms introduced in the Method section are used to improve the quality of these meshes. We test the initial and improved meshes in BEM computations of Poisson-Boltzmann (PB) electrostatics and generating corresponding surface conforming volume meshes that are required in FEM simulations. The BEM software used is a publicly available PB solver, AFMPB [37]. In FEM simulations, the corresponding volume meshes are generated by TetGen [38]. Table 1 shows some examples which demonstrate that our improvement algorithms are effective. The number of atoms of the test molecular ranges from several thousand to several hundred thousand. The PQR Benchmark can be downloaded from https://doi.org/10.6084/m9.figshare.5349229.v1. The initial molecular surface meshes are provided by TMSmesh 2.0. They are manifold meshes but contains highly irregular and singular triangles. The algorithms introduced in the Method section can remove the redundant points and improve the qualities of triangles effectively. The meshes are smoothed 100 rounds. The improved meshes can be applicable in AFMPB [37] and TetGen [38] directly. The volume mesh generated by TetGen can used in finite element method simulations, such as Ichannel [39], SMPBS [40], mFES [41] and so on.

**Table 1:** Results of applying the initial meshes and the improved meshes to AFMPB and TetGen.

Molecule	Natoms	Number of vertices		Number of triangles		AFMPB (solvation energy)		TetGen	
		Initial	Improved	Initial	Improved	Initial	Improved	Initial	Improved
AChE monomer	8280	67955	63361	135702	126782	-NAN	-2.50917e+03	Failed	Success
connexin	19884	213362	210219	426716	420778	-NAN	1.80728e+03	Failed	Success
AChe tetramer	36638	381665	366227	762514	732634	-NAN	-7.30957e+03	Failed	Success
30S ribosome	88431	489315	480332	979094	961920	-NAN	-2.14092e+06	Failed	Success
70S ribosome	165337	1160599	1147035	2324466	2298102	-NAN	-3.42853e+06	Failed	Success
3K1Q	203135	975340	925914	1948856	1853892	-NAN	-7.0773e+04	Failed	Success

Table 2 shows the CPU time for mesh quality improvement. All computations run on a computer with Intel^®^ Xeon^®^ CPU E5-4650 v2 2.4GHz and 126GB memory under 64bit Linux system. Before deleting the redundant points and doing Laplacian smoothing, we first check whether the mesh is manifold and delete the small cavities inside the molecules. If we reserve the small cavities, they tend to collapse after several times of Laplacian smoothing. The fourth column in Table 2 shows the time cost by checking manifoldness and deleting cavities. The fifth column shows the CPU time cost by deleting the redundant points and doing Laplacian smoothing.

**Table 2:** CPU time for mesh quality improvement by SMOPT.

Molecule	Number of vertices		CPU Time (s)	
	Initial	Improved	check manifold and delete small cavities inside	delete redundant points and do Laplacian smoothing
AChE monomer	67955	63361	3.39	43.06
connexin	213362	210219	21.04	201.52
AChe tetramer	381665	366227	59.22	364.55
30S ribosome	489315	480332	97.26	221.12
70S ribosome	1160599	1147035	447.80	580.70
3K1Q	975340	925914	359.49	465.84

**Fig 4:**
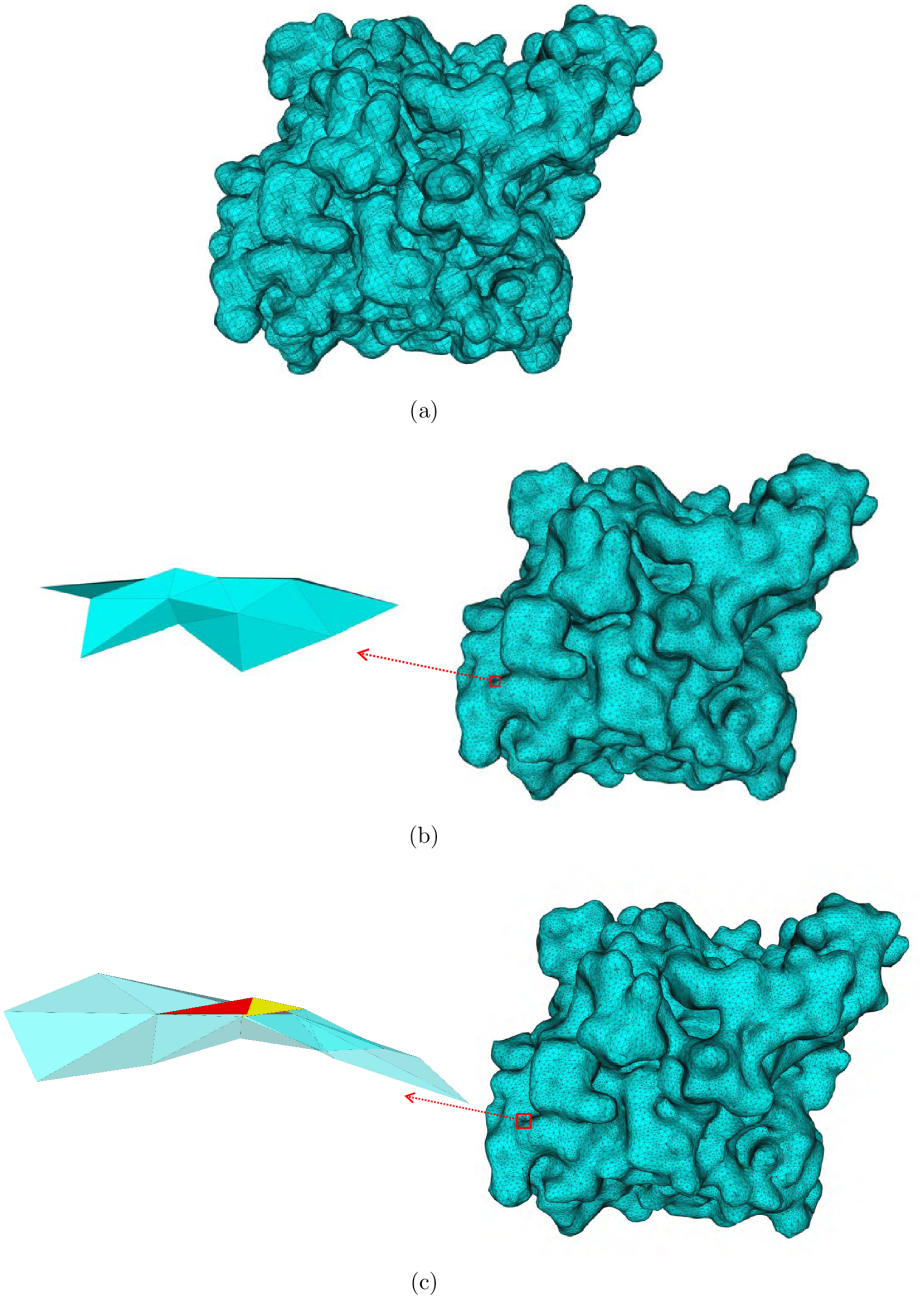
Initial mesh and improved meshes for an AChE monomer. (a) is the initial mesh, (b) is the improved mesh by SMOPT and (c) is the improved mesh by ISO2mesh. The left sub-figure in (c) shows an example of self-intersection in the mesh improved by ISO2mesh. The red triangle intersect with the yellow triangle. The left sub-figure in (b) shows the same region as the sub-figure in (c) and there is no intersection.

We compared the mesh quality of the improved meshes by our SMOPT with those by ISO2mesh [42]. ISO2mesh is a free matlab/octave-based mesh generation and processing toolbox. We use the meshresample and smoothsurf modules in ISO2mesh to improve the initial meshes. To make the improved meshes from ISO2mesh comparable to those from SMOPT, in the meshresample module, we set the vertex number in output meshes from ISO2mesh close to those from SMOPT. In the smoothsurf module of ISO2mesh, the Laplacian smoothing method is chosen and the meshes are smoothed 100 rounds. In addition, we also do the same test in MeshLab [43]. MeshLab is an open source system for processing and editing 3D triangular meshes. It provides a set of tools for editing, cleaning, healing, inspecting, rendering, texturing and converting meshes. We use MeshLab to do Laplacian smoothing for the meshes generated by TMSmesh 2.0. However, after several rounds of smoothing, some points with the coordinates (NaN, NaN, NaN) appear, which prevents us to do further test. So, here we only show the results from SMOPT and ISO2mesh. Fig 4 shows the initial and improved surface meshes by these two methods for an enzyme molecule AChE monomer. Compared to the initial mesh, both improved meshes are smoother and become closer to the molecular surface, and the two improved meshes are similar in the aspect of visualization. But there are self-intersections in the mesh improved by ISO2mesh. An example is shown in Fig 4. Table 3 compares mesh quality in aspect of manifoldness. There is no self-intersection in the meshes produced by SMOPT while the improved meshes by ISO2mesh can not preserve the manifoldness of the original meshes.

**Table 3:** Number of non-manifold errors in meshes produced by SMOPT and ISO2mesh.

Molecule	Number of vertices			Number of intersecting triangle pairs		
	Initial	Improved		Initial	Improved	
		SMOPT	ISO2mesh		SMOPT	ISO2mesh
AChE monomer	67955	63361	63341	0	0	57
connexin	213362	210219	207126	0	0	10
AChE tetramer	381665	366227	366262	0	0	256
30S ribosome	489315	480332	480503	0	0	317
70S ribosome	1160599	1147035	1146652	0	0	1316
3K1Q	975340	925914	925640	0	0	351

**Fig 5:**
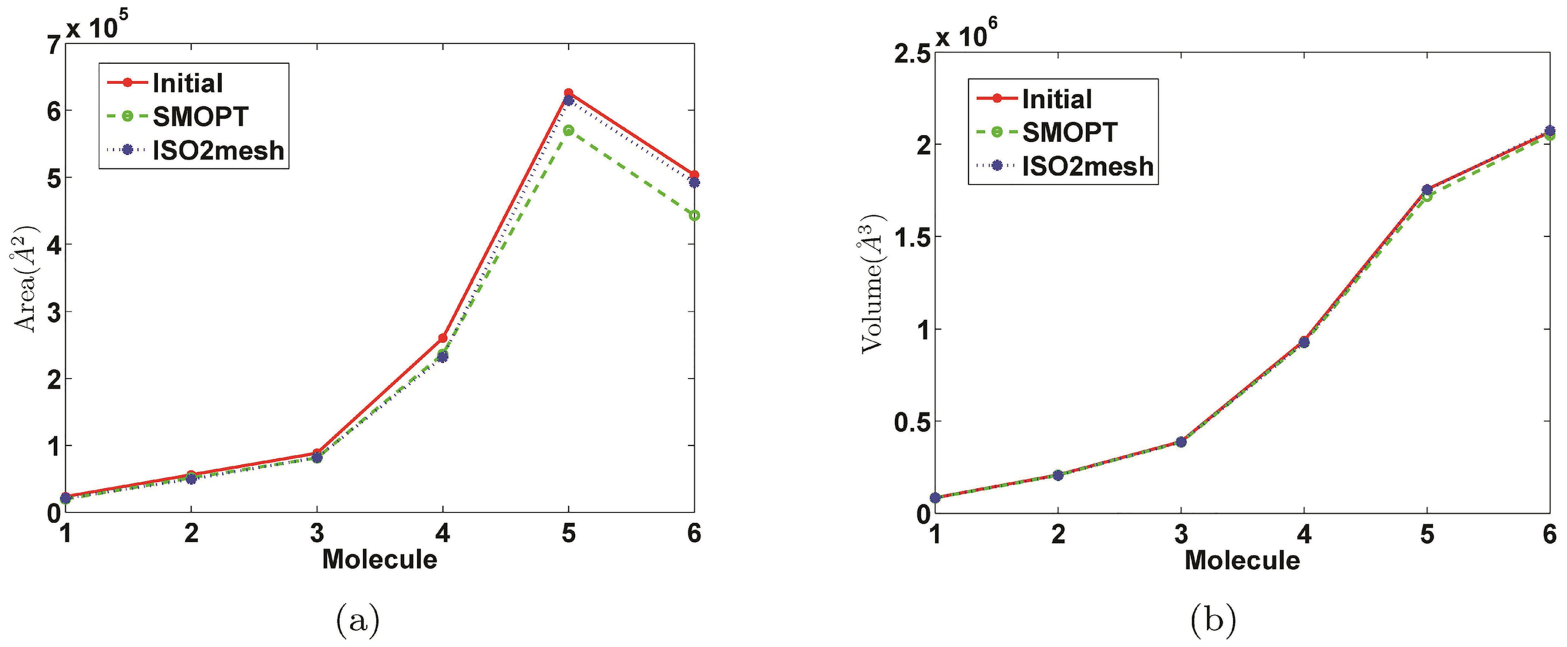
Comparison of area and volume between the initial meshes and the improved meshes generated by SMOPT and ISO2mesh for molecules in Table 3. (a) Area (b) Volume.

Fig 5 shows the areas and volumes of the initial meshes and the improved meshes generated by SMOPT and ISO2mesh. The areas of the improved meshes by SMOPT has small deviations from those of the initial meshes. Many roughed regions are smoothed after improvement, which will reduce the surface area. The volume of the smoothed mesh is almost preserved and keeps a deviation within 3%. ISO2mesh preserves both the mesh area and volume. An important reason is that ISO2mesh re-samples the vertices while SMOPT doesn’t. We will consider to add a re-sample module into SMOPT in the future.

**Fig 6:**
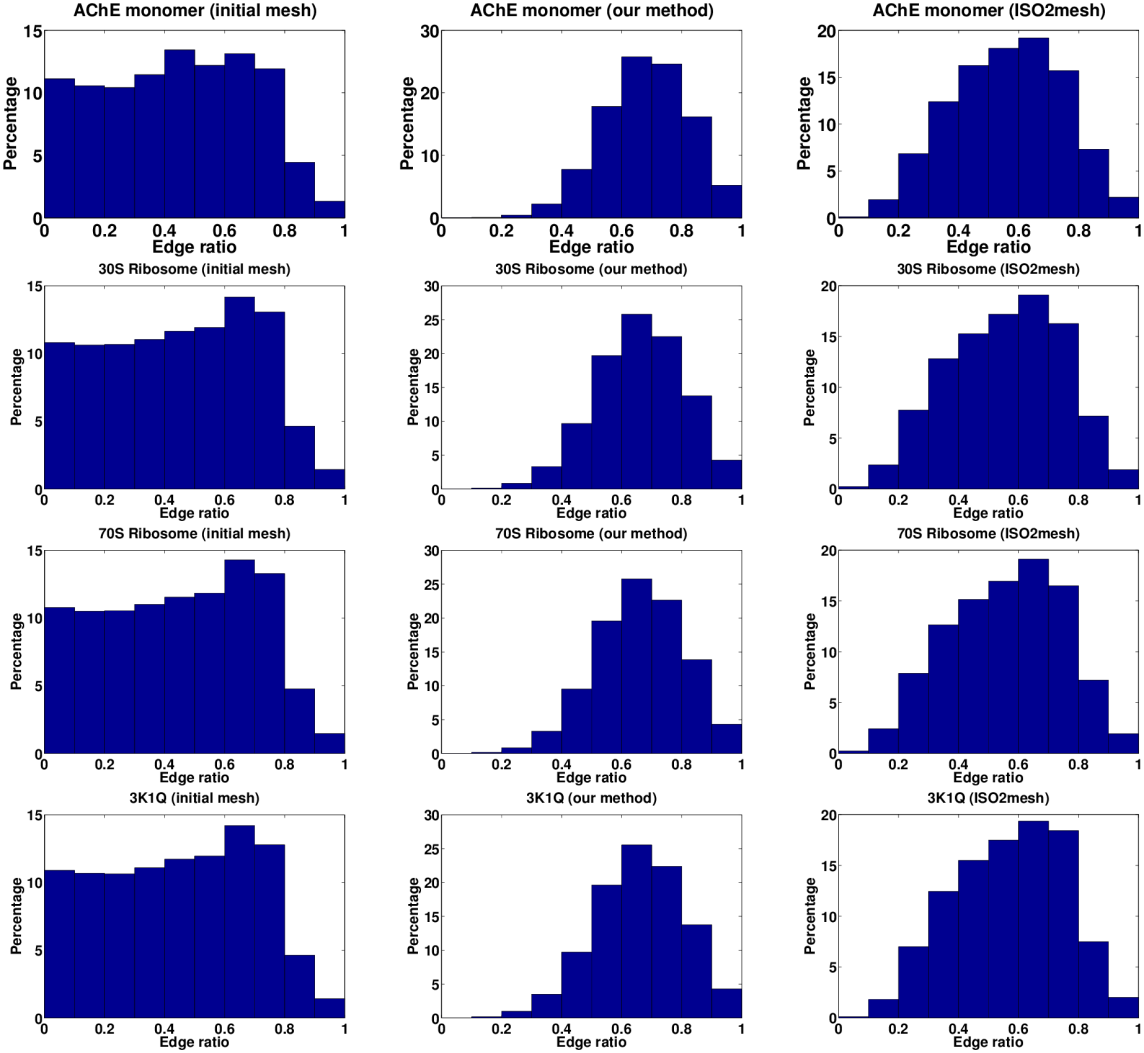
Distributions of ratio of the shortest edge length to the longest edge. The left column is the ratios of the initial mesh, the middle column is the ratios of the improved mesh by our method and the right column is the ratios of the improved mesh by ISO2mesh.

**Fig 7:**
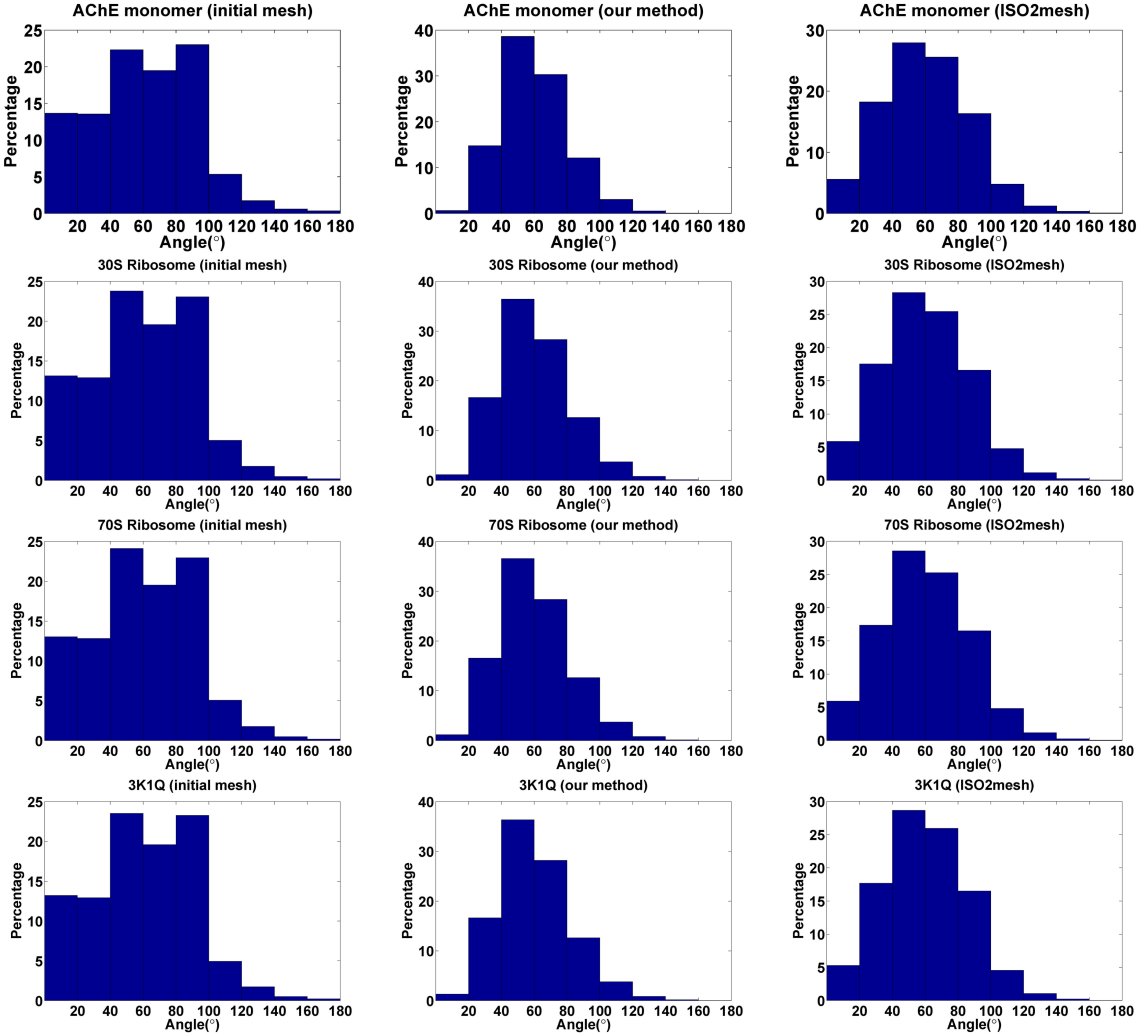
Distributions of angles of each triangle. The left column is the distributions of the initial mesh, the middle column is the distributions of the improved mesh by our method and the right column is the distributions of the improved mesh by ISO2mesh.

We also compared the uniformness of the meshes. The distribution of the ratios of the shortest edge length to the longest edge length of each triangle and the distribution of the angles of each triangle are used to describe the uniformness of a triangulated surface mesh. A ratio of 1.0 corresponds to an equilateral triangle, and a ratio close to 0 is indicative of a very poor uniformness. That is, the higher the ratio, the better the quality of the triangle. Fig 6 shows that the edge ratios of the initial meshes are uniform distributed between (0, 0.8). The edge ratios of the improved meshes by our method SMOPT and ISO2mesh are all clustered around 0.7, but the results by SMOPT are more concentrated than ISO2mesh. Additionally, there is no triangle whose edge ratio is between 0 and 0.1 in the improved meshes by our method, which means that there are few triangles with very poor quality after improvement. The angle distributions of surface triangles in the initial and improved meshes are shown in Fig 7. The angle distribution of the improved meshes are clustered around 40° to 80° and a small portion are close to 0° or 180°, which also indicates that the improved meshes have few sharp angles.

## Conclusion

In this paper, we have described a systematic procedure, SMOPT, to remove redundant points in a 3D triangular surface mesh and to smooth the mesh using a modified Laplacian smoothing method. The improved meshes are shown to be of good quality and can be applied to numerical simulation successfully. The program SMOPT can be used together with or integrated into a surface mesh generator, such as TMSmesh 2.0. SMOPT can be also used as a standalone software and is downloadable at https://doi.org/10.6084/m9.figshare.5346169.v1 and can be run online at http://www.xyzgate.com.
